# Immature Teratoma: A Case Report of a Monster Tumor in the Pediatric Age Group

**DOI:** 10.7759/cureus.48989

**Published:** 2023-11-18

**Authors:** Drashti Patel, Surekha Tayade, Sakshi Sharma, Lucky Srivani Reddy

**Affiliations:** 1 Obstetrics and Gynaecology, Jawaharlal Nehru Medical College, Datta Meghe Institute of Higher Education and Research, Wardha, IND

**Keywords:** fertility-sparing treatment, pediatric tumours, adjuvant chemotheapy, explorative laparotomy, unexpected ovarian malignancy, immature teratoma

## Abstract

Immature teratoma is a rare type of germ cell tumor containing embryonic tissues that may be malignant. It usually occurs in young women and affects the ovaries. Teratomas exhibit benign clinical behavior, but they can return as teratomas or with malignant components, and in a small subset of individuals, the prognosis may be deadly. We will discuss a case of a 9-year-old female child who presented with pain and a huge lump in the lower abdomen that was suggestive of an ovarian dermoid cyst or a germ cell tumor on computed tomography (CT) abdomen pelvis and underwent exploratory laparotomy and debulking surgery. Histopathology results indicated that she had a grade 3 immature teratoma. Postoperatively, the patient received 3 cycles of bleomycin, etoposide, and cisplatin (BEP) as adjuvant chemotherapy with a good response. She is currently under regular follow-up and has no evidence of recurrence or metastasis. This case illustrates the importance of early diagnosis and treatment of immature teratoma, which can be cured with surgery and chemotherapy. It also highlights the challenges of managing such a large tumor in a pediatric patient.

## Introduction

It is a tumor produced by an aberrant germ cell that divides during meiosis. There are two different forms of ovarian teratomas based on their histology: mature and immature. Mature ovarian teratoma, commonly known as a dermoid cyst, is the more frequent of these tumors. A mere one percent of all ovarian teratomas are immature, making them rare [1]. The term immature as a suffix with tumor denotes incomplete cell differentiation which reveals more antagonistic action as 25 to 30% mortality between 10 to 20 years of age group is due to ovarian cancer. It contains immature tissue generated from the ectoderm, mesoderm, and endoderm, three embryologic layers. They frequently surpass mature cystic teratomas in size [1]. The Greek word "teraton" (which means a monster) is where the word "teratoma" originates. Teratomas exhibit benign clinical behavior, but they can return as teratomas or with malignant components, and in a small subset of individuals, the prognosis may be deadly [2]. The tumors originate from totipotent cells that have lost some of their genetic code. Ovarian teratoma (OT) is effective in up to 15-year-old children. Sacrococcygeal teratoma (SCT) is proven effective in neonates with a female preponderance. Both developed and undeveloped parts are considered non-cancerous cells, and the possibility of metastasis is unknown [2,3]. Mostly hard and lobulated, undeveloped teratomas have several tiny cysts. Juvenile bone and cartilage may be apparent in solid portions. Observation of a variety of tumors can describe differentiation into nerves (neuroepithelial rosettes and immature glia), glands, and other structures found in mature cystic teratomas. Nearly one-third of immature teratomas express serum-fetoprotein [3]. Immature ovarian teratomas are usually found in girls up to their early 20s. Even if these teratomas are diagnosed at an advanced stage, most cases are cured by a combination of surgery and chemotherapy. Serum-fetoprotein is expressed by nearly one-third of immature teratomas [3]. The long-term outlook of individuals with immature teratomas is determined by the tumor's stage (International Federation of Gynecology and Obstetrics, FIGO) and grade. The tumor's grade is determined by the immaturity of the different tissues. Grade 3 tumors are made up of the most immature tissues, with a large proportion of embryonic neuroepithelium [3]. 

## Case presentation

**Figure 1:**
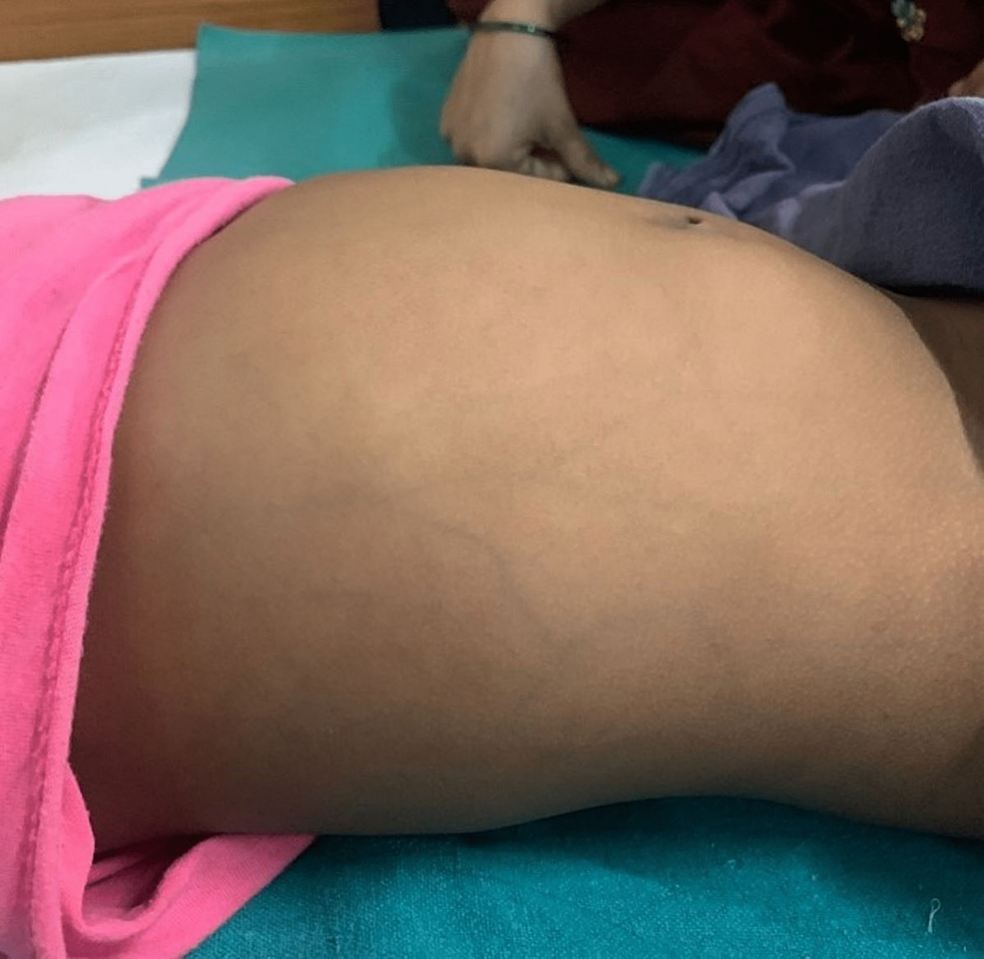
Clinical photograph showing a mass of approximately 12-14 weeks in size which extended till the umbilicus.

A 9-year-old female child presented to a rural tertiary care hospital with complaints of vomiting and pain in the abdomen persisting for the past 1 month. There was no change in bladder habits or bowel. On general examination, she was visibly pale. On abdominal examination, a mass of approximately 10*10 cm in size extended to the umbilicus, and veins were visible to the abdominal wall, which was hard in consistency, and immobile (Figure 1).

The patient had elevated levels of serum markers suggestive of a germ cell tumor. Her alpha-fetoprotein (AFP) was 5500 ng/mL, which is much higher than the normal range of less than 40 ng/mL. Her beta-human chorionic gonadotropin (B-HCG) was 66.85 mIU/mL, which is slightly above the normal range of 10-50 mIU/mL. Her CA-125 was 56.2 U/mL, which is also above the normal range of less than 35 U/mL. Her CA-19.9 and CEA were within the normal ranges, at 14 U/mL and 2.48 ng/mL, respectively. Her lactate dehydrogenase (LDH) was elevated at 388 U/L, compared to the normal range of 130-214 U/L. Based on these findings, a clinical diagnosis of a germ cell tumor was made.

**Figure 2:**
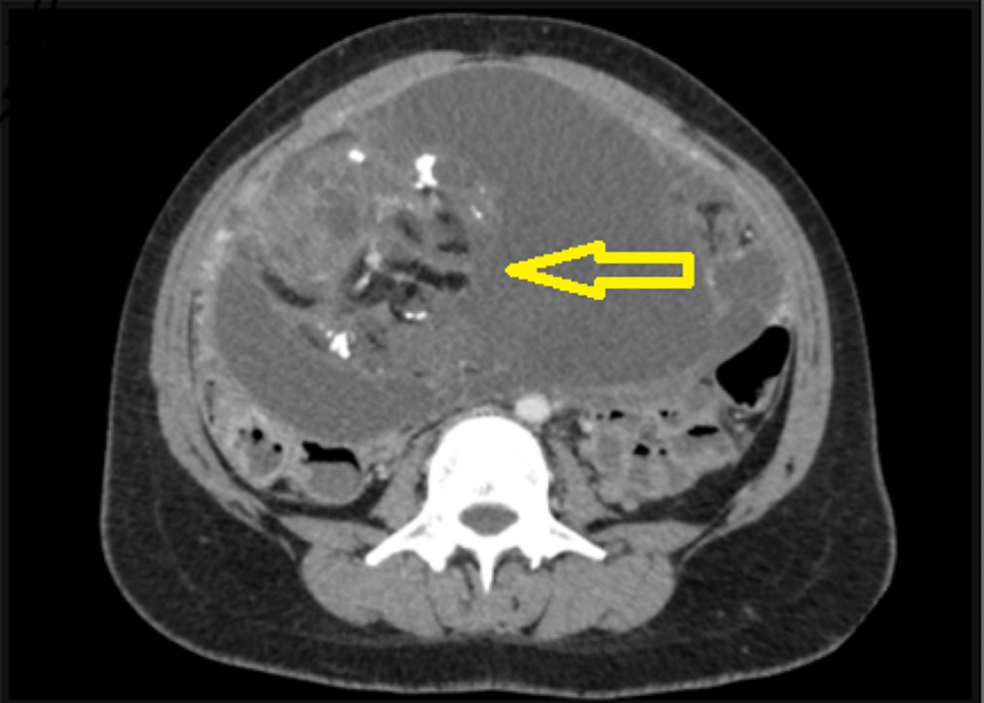
Contrast-enhanced computed tomography of the abdomen and pelvis showed a large well-defined heterogeneously enhancing lesion of size 85 x 75 x 90 mm in the lower abdominopelvic region. The lesion shows fat density, solid components, and calcific foci within it.

No sonography or X-ray was done in the past. A contrast-enhanced CT scan of the abdomen and pelvis revealed a large, heterogeneous mass in the lower abdominopelvic region, measuring 85 x 75 x 90 mm. The mass had features of fat density, solid components, and calcifications. The bilateral ovaries and the uterus could not be seen separately from the mass, indicating that they were involved in the tumor. These findings were consistent with a germ cell tumor (Figure 2).

**Figure 3:**
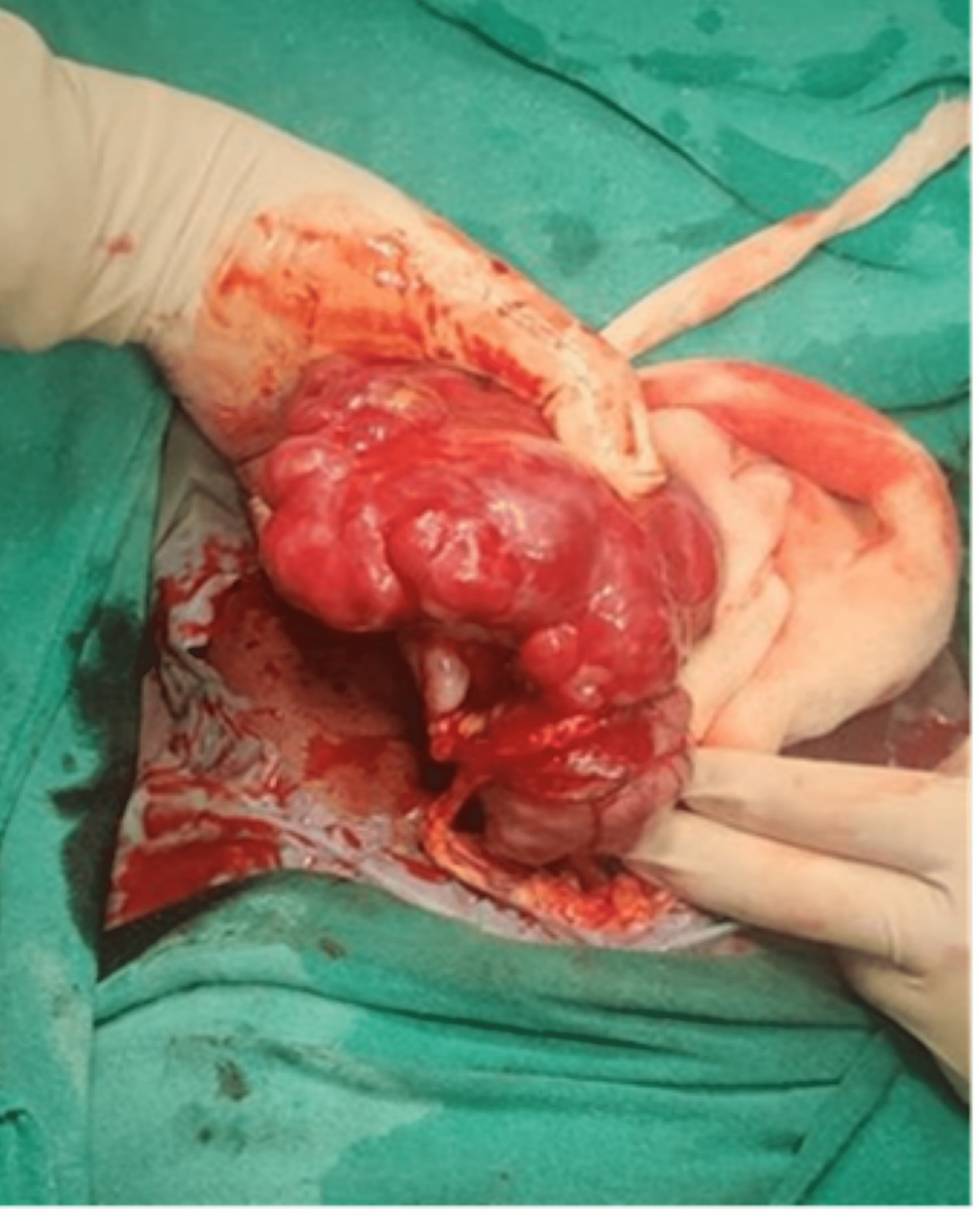
An intraoperative right ovarian lobulated mass of approximately 9 x 6 x 8 cm in size which was hard, and irregular with the capsule intact was found.

**Figure 4:**
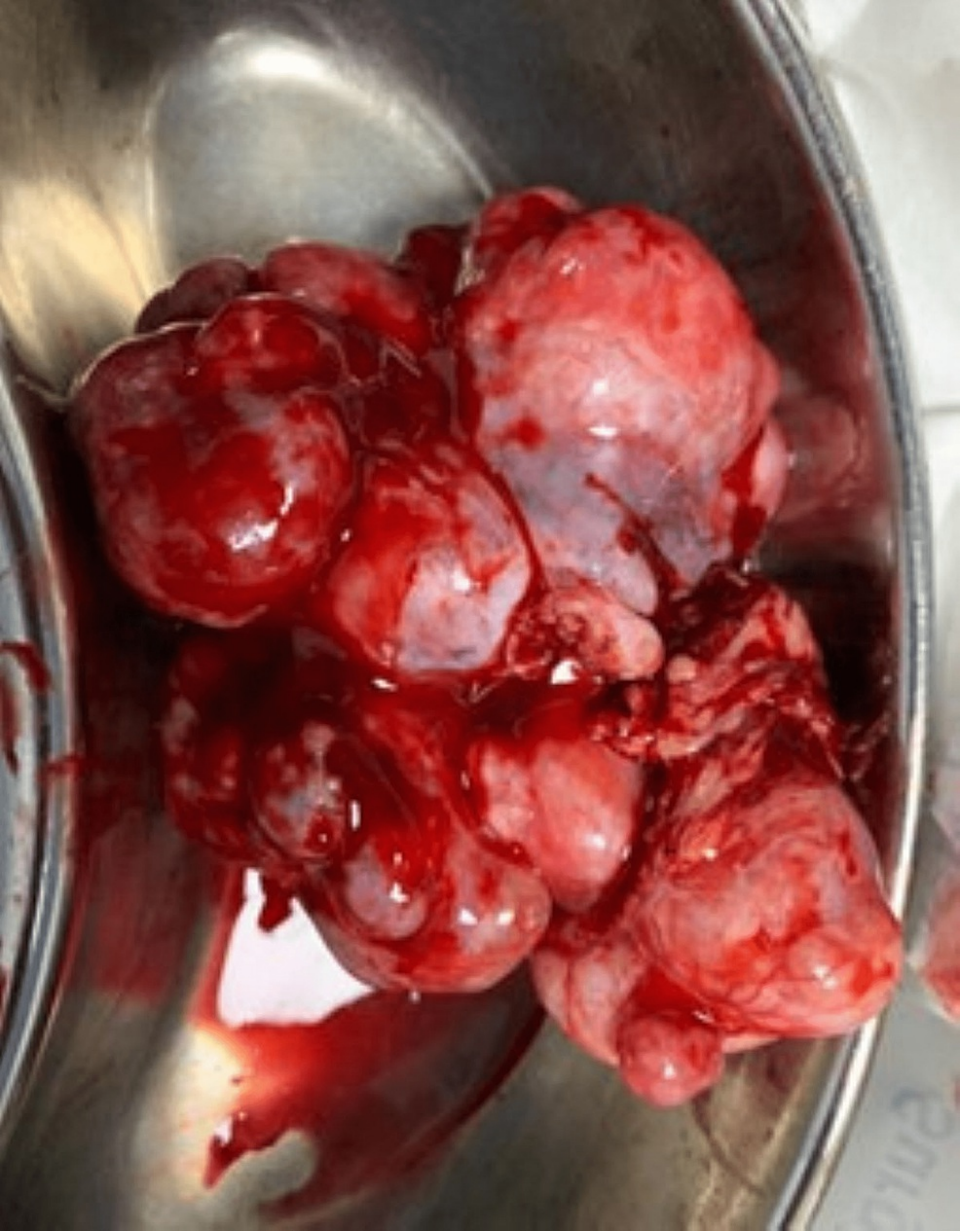
Right ovarian lobulated mass removed during exploratory laparotomy.

**Figure 5:**
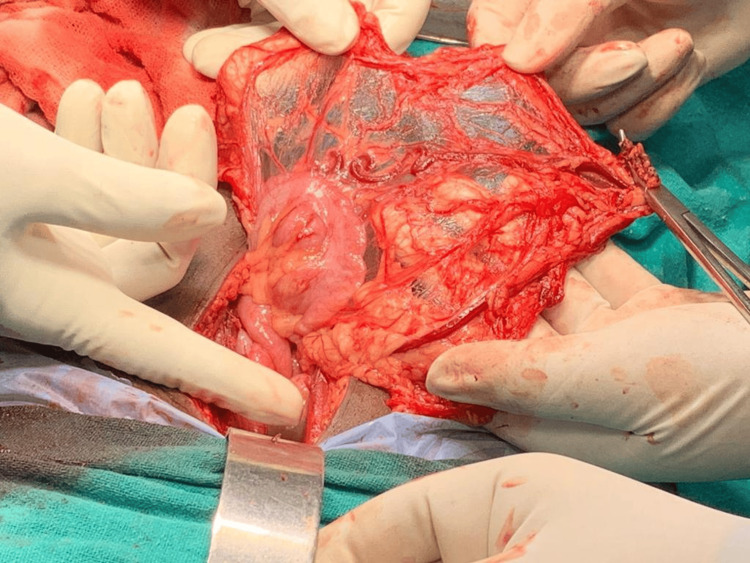
Intraoperative infracolic omentectomy.

**Figure 6:**
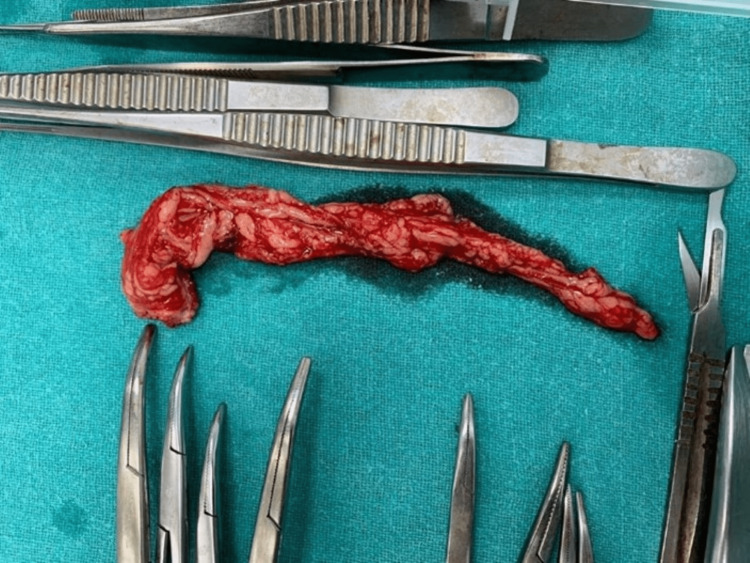
Infracolic omentum removed.

**Figure 7:**
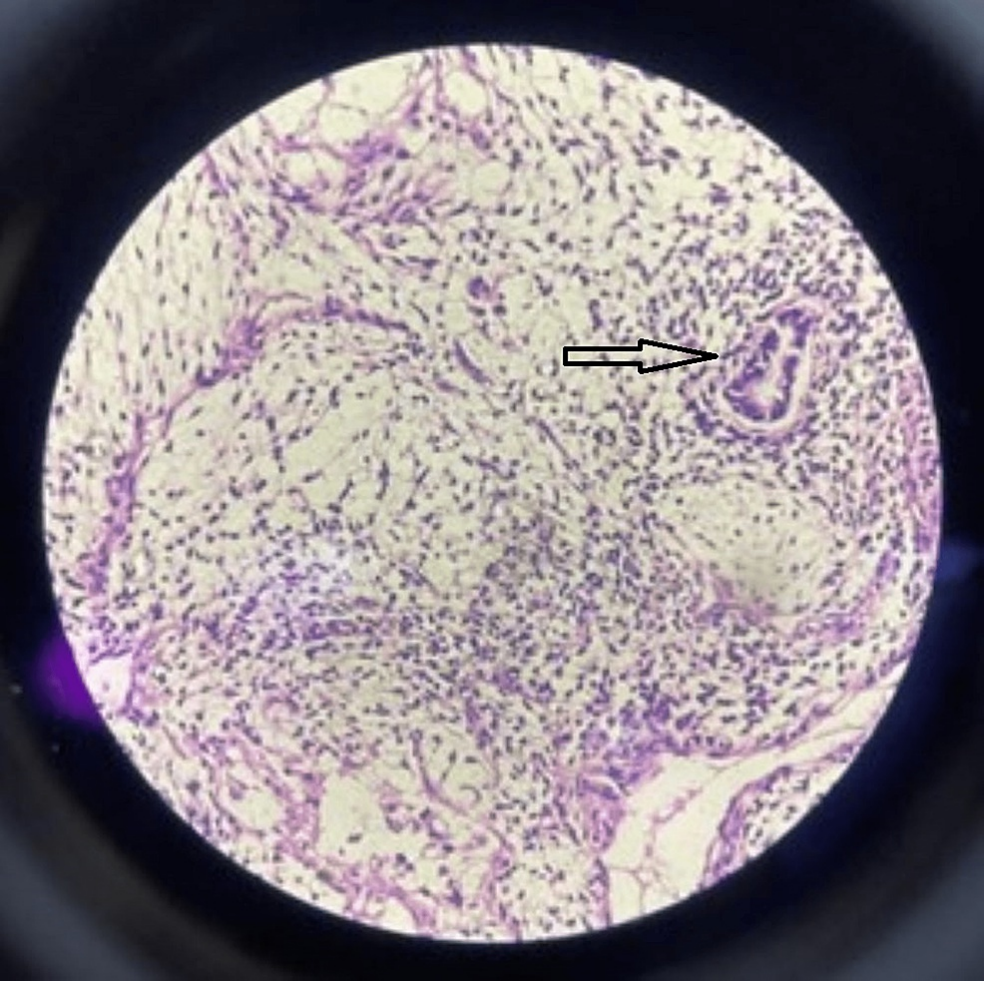
Histopathology (40x) (H & E stain) showing immature neuroectodermal tissue with rosettes. H & E: hematoxylin and eosin

**Figure 8:**
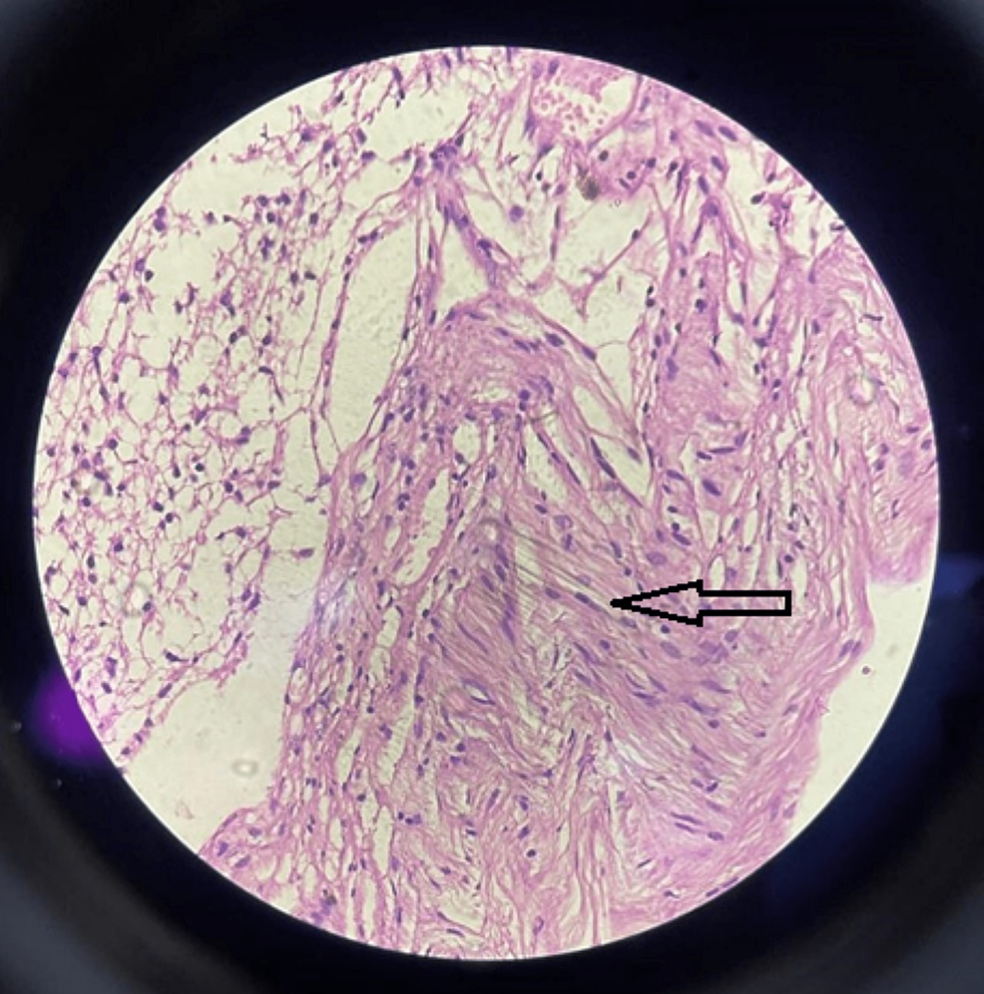
Histopathology (10x) (H & E stain) showing characteristic spindle-shaped cells suggestive of immature teratoma. H & E: hematoxylin and eosin

**Figure 9:**
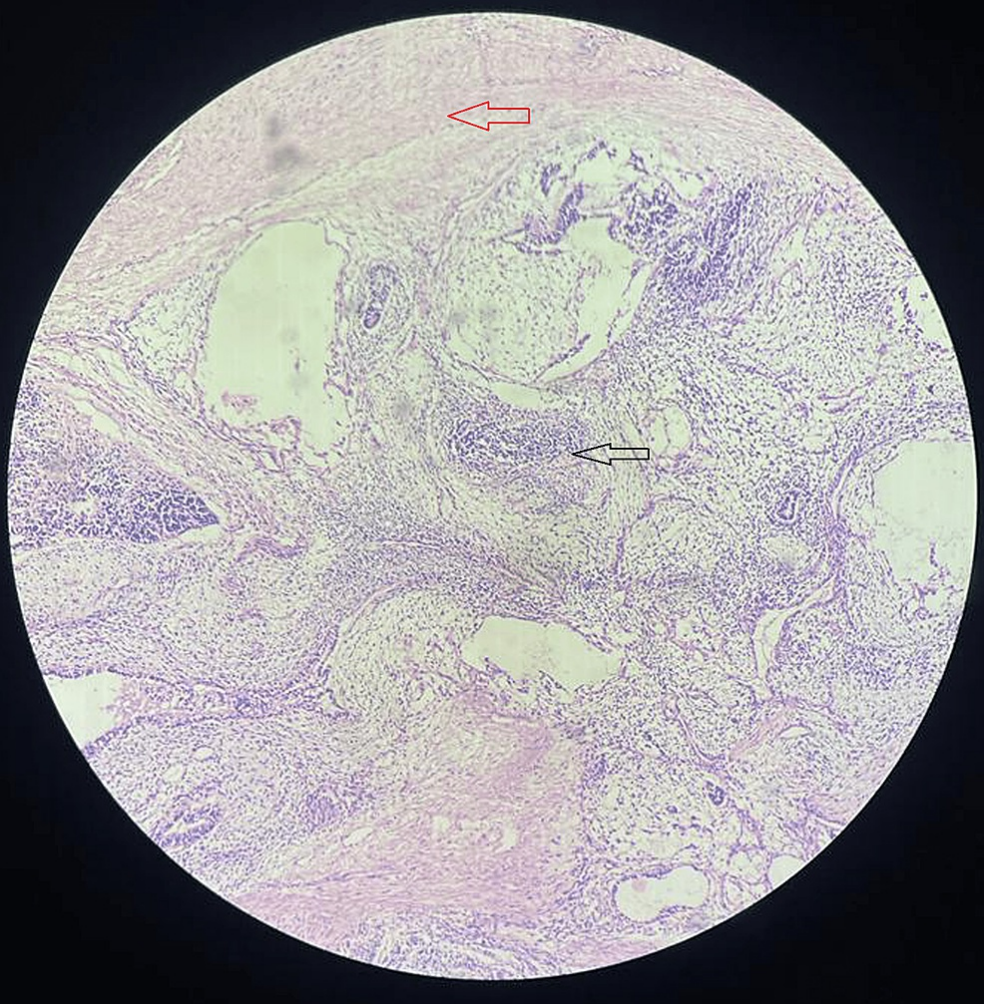
Histopathology (4x) (H & E stain) showing predominantly fibro collagenous and fibro adipose tissue (red arrow) along with glandular tissue lived with tall columnar epithelium and neural tissue comprising ganglion cell and lymphocytic aggregates (black arrow) and blood vessels at places. H & E: hematoxylin and eosin

Exploratory laparotomy and debulking surgery were done in which approximately 9 x 6 x 8 cm right ovarian lobulated mass, which was hard and irregular with the capsule intact, was removed; the surgery was debulking due to the hard ovarian mass (Figures 3, 4) and sent for frozen section, and the histopathology report suggested immature teratoma. The omentum was inspected for any metastasis, and an infracolic omentectomy was done (Figures 5-9).

Further postoperatively, tumor board discussions including a pediatrician were carried out with a team comprising a gynecologist, a physician, a surgeon, a pathologist, and a radiotherapist. As the disease was immature teratoma grade 3, it was decided that she should receive 3 cycles of bleomycin, etoposide, and cisplatin (BEP) and advised to follow up every 3 months for one year.

The patient received postoperative adjuvant chemotherapy of 3 cycles of BEP. In each cycle, she received bleomycin 15 international units of on day 1, day 8, and day 15; cisplatin 18 milligrams of on day 1 to day 5; and etoposide 90 milligrams of on day 1 to day 5 and responded well to the treatment. The patient developed side effects such as vomiting, constipation, hair loss, and gastritis. The patient was followed up on an outpatient basis every 3 months for a year and 6 monthly for another consecutive year. No tumor was found in the sonography examination. AFP level was normal.

## Discussion

Immature teratoma, a rare type of germ cell tumor, presents unique challenges, particularly when encountered in the pediatric age group. This case report highlights the clinical presentation, diagnostic approach, and multidisciplinary management of a 9-year-old female with a large immature teratoma. The clinical presentation of immature teratoma often includes nonspecific symptoms, making early diagnosis challenging. In this case, the patient presented with vomiting, abdominal pain, and a palpable mass. The elevation of serum tumor markers, including AFP, beta-human chorionic gonadotropin (B-HCG), and CA-125 played a crucial role in raising suspicion of a germ cell tumor. Similar findings have been reported in the literature, emphasizing the significance of tumor markers in the diagnosis and monitoring of germ cell tumors [4].

Imaging studies, particularly contrast-enhanced computed tomography (CT), provided essential information for surgical planning. The heterogeneous nature of the tumor, including features such as fat density, solid components, and calcifications, aligns with previous literature on the radiological characteristics of immature teratomas [5]. Imaging modalities are pivotal in guiding surgical intervention and assessing the extent of tumor involvement.

Surgical resection remains the cornerstone of treatment for immature teratomas. The presented case underwent exploratory laparotomy and debulking surgery, leading to the removal of the ovarian mass [6]. Histopathological examination confirmed the diagnosis of grade 3 immature teratoma, emphasizing the necessity of surgical intervention for both diagnostic and therapeutic purposes. Surgical outcomes are consistent with studies advocating for aggressive surgical management in cases of immature teratomas [7].

Adjuvant chemotherapy, specifically the combination of bleomycin, etoposide, and cisplatin, has shown efficacy in treating germ cell tumors. The decision to administer adjuvant chemotherapy, in this case, was based on the tumor's grade and the risk of recurrence. The patient responded well to the treatment, experiencing expected side effects. Similar chemotherapy regimens have demonstrated success in previous studies, supporting their role in managing immature teratomas [8].

Long-term follow-up is essential for monitoring treatment efficacy and detecting potential recurrence. In this case, regular follow-ups, including sonography examinations and tumor marker assessments, revealed no evidence of recurrence, indicating a favorable outcome. Long-term surveillance aligns with the literature, emphasizing the importance of extended follow-up periods for patients with germ cell tumors [9].

The multidisciplinary approach, as demonstrated by tumor board discussions involving specialists from various fields, is critical in managing complex cases. Collaboration among pediatricians, gynecologists, surgeons, pathologists, and radiotherapists ensures comprehensive and well-informed decision-making. This approach is consistent with current recommendations for managing germ cell tumors, underscoring the importance of a team-based approach [10].

## Conclusions

Immature teratoma is a rare and potentially malignant germ cell tumor that can affect young girls and women. It can present with nonspecific symptoms such as abdominal pain and distension and can grow very large. The diagnosis is based on clinical features, serum markers, and imaging studies. The treatment of choice is the surgical removal of the tumor, followed by chemotherapy in high-grade or metastatic disease cases. The prognosis is generally good, especially if the tumor is detected and treated early. Fertility preservation is an important factor to take into account while treating immature teratomas. For some patients with early-stage or low-risk tumors, fertility-sparing surgery may be an option. This case report demonstrates the successful management of a 9-year-old girl with a huge immature teratoma involving her left ovary, who achieved complete remission after surgery and chemotherapy.
